# Dynamics of soluble and cellular inflammatory markers in nasal lavage obtained from Cystic Fibrosis patients during intravenous antibiotic treatment

**DOI:** 10.1186/1471-2466-14-82

**Published:** 2014-05-13

**Authors:** Julia Hentschel, Manuela Jäger, Natalie Beiersdorf, Nele Fischer, Franziska Doht, Ruth K Michl, Thomas Lehmann, Udo R Markert, Klas Böer, Peter M Keller, Mathias W Pletz, Jochen G Mainz

**Affiliations:** 1 CF-Centre, Pediatrics, Jena University Hospital, Jena, Germany; 2 Institute of Medical Statistics, Computer Sciences and Documentation, Jena University Hospital, Jena, Germany; 3 Department of Obstetrics, Placenta Laboratory, Jena University Hospital, Jena, Germany; 4 Institute for Clinical Chemistry and Laboratory Diagnostics, Jena University Hospital, Jena, Germany; 5 Institute of Medical Microbiology, University of Jena, Jena, Germany; 6 Center for Infectious Diseases and Infection Control, Jena University Hospital, Jena, Germany

**Keywords:** Cystic Fibrosis, Paediatric pulmonology, Upper airways (UAW), Nasal lavage, Inflammation, Cytokines, Antibiotic treatment, Permanent UAW colonization, Cytology

## Abstract

**Background:**

In cystic fibrosis (CF) patients, the upper airways display the same ion channel defect as evident in the lungs, resulting in chronic inflammation and infection. Recognition of the sinonasal area as a site of first and persistent infection with pathogens, such as *Pseudomonas aeruginosa*, reinforces the “one-airway” hypothesis. Therefore, we assessed the effect of systemic antibiotics against pulmonary pathogens on sinonasal inflammation.

**Methods:**

Nasal lavage fluid (NLF) from 17 CF patients was longitudinally collected prior to and during elective intravenous (i.v.) antibiotic treatment to reduce pathogen burden and resulting inflammation (median treatment time at time of analysis: 6 days). Samples were assessed microbiologically and cytologically. Cytokine and chemokine expression was measured by Cytometric Bead Array and ELISA (interleukin (IL)-1β, IL-6, IL-8, MPO, MMP9, RANTES and NE). Findings were compared with inflammatory markers from NLF obtained from 52 healthy controls.

**Results:**

Initially, the total cell count of the NLF was significantly higher in CF patients than in controls. However after i.v. antibiotic treatment it decreased to a normal level. Compared with controls, detection frequencies and absolute concentrations of MPO, IL-8, IL-6 and IL-1β were also significantly higher in CF patients. The detection frequency of TNF was also higher. Furthermore, during i.v. therapy sinonasal concentrations of IL-6 decreased significantly (*P* = 0.0059), while RANTES and MMP9 levels decreased 10-fold and two-fold, respectively. PMN-Elastase, assessed for the first time in NFL, did not change during therapy.

**Conclusions:**

Analysis of NLF inflammatory markers revealed considerable differences between controls and CF patients, with significant changes during systemic i.v. AB treatment within just 6 days. Thus, our data support further investigation into the collection of samples from the epithelial surface of the upper airways by nasal lavage as a potential diagnostic and research tool.

## Background

Cystic fibrosis (CF) is the most common autosomal recessive disorder in the Caucasian population and is caused by mutations in the Cystic Fibrosis Transmembrane Conductance Regulator (*CFTR*) gene (chromosomal position 7q31.2), leading to altered chloride ion exchange and hyperviscous mucus in the affected organs. Patients also suffer from recurrent infections of the respiratory tract and chronic inflammation, which leads to tissue remodelling and finally to premature death caused by respiratory insufficiency [1]. The causative *CFTR* defect also affects sinonasal mucosa, so that almost 100% of CF patients reveal a pathological sinonasal computer tomography [2]. In addition to impairing the patient’s quality of life, the involvement of the upper airways (UAW) in CF has the potential to aggravate the overall course of disease. Most importantly, sinonasal involvement in CF facilitates *de novo* and persistent airway colonisation with pathogens including *Pseudomonas (P.) aeruginosa*[3,4], which is the major cause of morbidity and mortality. Thus, cross-colonisation between the airway levels is evident as *P. aeruginosa* strains in sputum and UAW specimens in patients who harbour the pathogen in both airway levels are genetically identical [4-6]. Additionally, the paranasal sinuses have been identified both as a site for the diversification of *P. aeruginosa* before spreading into the lower airways [7] and as a site of persistence in CF patients who underwent lung transplantation, whereupon these clones colonise the transplanted lungs that were primarily free from *P. aeruginosa*[8]. It was shown that sinus surgery together with an intensive antibiotic follow-up treatment, as well as conservative methods such as sinonasal inhalation using vibrating aerosols, can eradicate *P. aeruginosa* from the upper airways and so decrease pulmonary infection events [3,9]. Therefore, it is very important to recognize the upper and lower airways as a “one-airway system” and not neglect the upper airways in the routine care of CF patients [10].

Nasal lavage (NL), which is frequently used in the field of allergies, e.g. for monitoring of provocation effects [11], is the most sensitive method for non-invasive assessment of pathogen colonisation of the UAWs [4].

Polymorphonuclear leukocytes (PMN) are major players in the first line of defence against pathogens. Most proteases and cytokines important for host defence and inflammation are released by neutrophil cells. Pulmonary secretions from CF patients obtained by bronchoalveolar lavage (BAL) revealed elevated levels of interleukin (IL)-1β, IL-6 and TNF, especially in patients infected with *P. aeruginosa*[12,13]. Moreover, recently Paats et al. reported significantly elevated IL-6 concentrations in the NL of CF patients during acute exacerbations, compared with controls 3 months later. Systemic IL-6 levels correlated significantly to several clinical parameters in both stages of disease. In our previous study assessing the NL of CF patients [14], IL-1β and IL-6 were detected more frequently in CF compared with healthy controls. In contrast, TNF that had been elevated in the BAL of CF patients was not detectable in the NL-fluid (NLF) of CF and healthy controls [14]. However, especially for IL-1β, IL-6 and TNF, differences in cytokine expression between upper and lower airways and peripheral blood were observed, suggesting a compartmentalised local inflammatory response [15,16]. IL-8 encourages neutrophils to leave the circulation and migrate into the tissue. In the NLF of CF patients, IL-8 was detected more frequently than in healthy controls [14]. An increase of PMNs and IL-8 in the upper airways of CF patients has also been reported [17]. IL-8 mRNA expression was increased in CF patients [18], and IL-8 levels in UAW and in the lower airways (LAW) showed a significant correlation [19].

Myeloperoxidase (MPO) is produced by stimulated neutrophils and catalyses the production of various oxidants [20,21]. Elevated MPO is an established marker for neutrophil activity as it is released in oxidative bursts. In the recent work by Beiersdorf et al. [14], MPO was elevated in the NL of CF patients compared with healthy controls. Matrix metalloproteinase 9 (MMP9), also produced by neutrophils, is involved in the breakdown of extracellular matrix proteins such as elastin or collagen [22]. MMPs are physiologically cleaved by tissue inhibitors of metalloproteinases (TIMPs) and are involved in physiological processes including tissue remodelling, but also in pathological processes when their balance is disturbed. In the literature, the role of the serine protease neutrophil elastase (NE), which is released on stimulation with TNF or IL-8 [23], has been intensively studied in the lower airways of CF patients, but little is known of its relevance in the upper airways. Normally, NE plays an important role in the processing and release of cytokines (e.g. IL-6 [24]), modulation of immune cell activity and mucus secretion [25]. It is also important in the defence against gram negative bacteria including *P. aeruginosa* by cleaving bacterial cell surface structures, such as flagella. In the CF lung, NE is over-expressed leading to dysfunction of the innate and adaptive immune systems. RANTES/CCL5 (regulated on activation, normal T cell expressed and secreted) is a chemoattractant that recruits and activates eosinophils and this was shown to be elevated in nasal polyps of CF patients [26].

Non-invasively collected NL from the patient’s UAW epithelial lining fluid can open the field to monitor airway colonisation, host defence and inflammation, which has rarely been considered in the recent literature. In particular, monitoring inflammatory mediators in NLF during interventions as a non-invasive outcome parameter requires further investigation. The aim of the present study is to assess changes in leukocyte populations and expression of IL-1β, IL-6, IL-8, TNF, RANTES, MPO, MMP9 and NE in the upper airways of CF patients during systemic antibiotic (AB) treatment to establish a better understanding of inflammation and immune defence mechanisms. The findings were also compared with inflammatory markers in NLF from a healthy control group.

## Methods

### Study population

Paediatric and adult patients, diagnosed with CF by two sweat tests and/or detection of two causative *CFTR*-mutations, who electively received systemic i.v. AB treatment, were prospectively included in the study at the CF Centre, Jena University Hospital, between August and December 2010. All patients were chronically colonised with high-risk pathogens. They were treated electively with i.v. AB, according to a standard used in many European CF Centres [27] to reduce pathogen burden, inflammatory responses and pulmonary destruction. The inclusion criteria were the ability to perform NL (see below) and be CF patients receiving elective i.v. AB treatment as part of their routine care. The exclusion criteria were perforation of tympanic membranes and a previously initiated systemic AB therapy within the previous 2 weeks. Only azithromycin (AZM) therapy was allowed and documented.

Sinonasal samples were taken directly before and during/after i.v. therapy. Chronic rhinosinusitis (CRS) in CF patients was diagnosed according to the European position paper on rhinosinusitis and nasal polyps 2012 (EPOS) [28]. Clinical data (e.g. lung function, systemic inflammation) were assessed only before, and not after, AB treatment as patients completed treatment at home.

The 52 healthy controls were recruited as described previously [14].

#### Ethics statement

All patients (or parents of minors) gave their written informed consent. The study was approved by the Ethics Committee of the Jena University Hospital.

### Nasal lavage (NL)

#### Sampling

NL was performed as previously described [4,29] using 10 mL of sterile isotonic saline (NaCl) for each nostril. Backflow was rinsed into a sterile plastic cup supported by the patient breathing out lightly.

#### NL processing

Recovered volumes were measured before aliquoting. An aliquot was directly sent for microbiological analysis (see below). Another aliquot was used for cytological analysis after stabilising cells in 10% foetal calf serum (FCS, Biochrom, Berlin, Germany). The lavage sample was centrifuged (160×g, 10 min, RT), supernatant discarded and the pellet resuspended in 1 mL 0.9% NaCl supplemented with 10% FCS. The remaining volume of NLF was divided; one part was stored without centrifugation (natively) at -80°C, the other one was centrifuged (160×g, 10 min, RT), and supernatant was frozen at -80°C within 45 minutes of sampling. A protease inhibitor cocktail (Protease Inhibitor Mix G, Serva, Germany) was added to each aliquot prior to freezing. The protein concentration was measured in single assays at a wavelength of 280 nm using a NanoDrop ND 1000 spectrophotometer (Thermo Fisher Scientific Inc., Waltham, MA, USA).

### Cytological analysis

Analysis of the total cell count and the automated differentiation of cells was performed using a XE-5000 haemocytometer (Sysmex, Norderstedt, Germany) in Body Fluid Modus. For cytological differentiation, cytospin preparations of 100 cells were prepared.

#### Microbiological analysis

Microbiological analyses were performed according to European standards [30]. Permanent and intermittent colonisation was determined using the criteria published by Lee et al. [31], where chronic colonisation is when more than 50% of cultures within the preceding year are positive and intermittent colonisation is if less than 50% of cultures are positive for a given pathogen.

### Immunological analysis

#### Cytometric bead array and FACS analysis

Analysis of MMP9, MPO, RANTES, IL-1β, IL-6, IL-8 and TNF was carried out using a Cytometric Bead Array (FlowCytomix, eBioscience, San Diego, CA, USA) followed by flow cytometry (FACS Calibur, BD, Franklin Lakes, NJ, USA) as described elsewhere [14]. The results were evaluated using FlowCytomix Pro 2.3 software (eBioscience, Frankfurt, Germany). Bead array experiments were done in single assays. Table 1 provides details of the detection limits.

**Table 1:** Inflammation markers in controls and CF patients before and during AB treatment

**Analyte**	**DL**	**Detection frequency (%)**				**Inflammatory marker concentration**						
						**Median**			**Range**			***P***
		**Controls**	**CF prior therapy**	**CF during therapy**	***P***	**Controls**	**CF before therapy**	**CF during therapy**	**Controls**	**CF before therapy**	**CF during therapy**	
MMP9 (ng/mL)	0.095	n.m.	100.0	100.0	---	n.m.	11.4	5.5	n.m.	2.02-122.2	1.8-25.2	0.0523^‡^
MPO (ng/mL)	0.2	71.2	100.0	100.0	0.0008^◊†^	33.4	215.3	171.9	0.1-182.7	54.36-531.1	21.4-533.4	0.0008^◊†^
												0.7404^‡^
IL-8 (pg/mL)	0.5	61.5	94.1	88.2	0.0008^◊†^	92.6	1145.3	756.1	0.4-11514.8	0.4-1976.7	0.4-2265.6	0.0008^◊†^
					0.7728^‡^							0.4488^‡^
IL-6 (pg/mL)	1.2	1.9	60.0	46.7	0.0008^◊†^	0.1	45.1	1.1	1.1-80.2	1.1-104.9	1.1-40.2	0.0008^◊†^
					0.4237^‡^							0.0059^‡^
IL-1β (pg/mL)	4.2	3.8	73.3	60.0	0.0008^◊†^	4.1	174.5	130.6	4.1-152.8	4.1-779.3	4.1-1052.8	0.0008^◊†^
					0.3428^‡^							0.8311^‡^
TNF (pg/mL)	3.2	15.4	60.0	66.7	0.0008^◊†^	3.1	55.4	55.4	3.1-46.6	3.1-1036.0	3.1-242.5	0.6416^◊†^
					0.7656^‡^							0.5693^‡^
RANTES (pg/mL)	25.0	n.m.	73.3	53.3	0.1294^‡^	n.m.	287.7	25.0	n.m.	25.0-1612.0	25.0-646.4	0.0942^‡^
NE (ng/mL)	0.16	n.m.	100.0	100.0	---	n.m.	0.9	0.8	n.m.	0.27-5.0	0.3-11.4	0.9382^‡^

^◊^*P*-value between controls and CF before therapy (after Bonferroni adjustment), ^†^*P*-value between controls and CF during therapy (after Bonferroni adjustment), ^‡^*P*-value between CF before and during therapy. *DL* = Detection Limit, *n.m.* = not measured.

#### ELISA

NL PMN-Elastase (NE) concentrations were determined in duplicates of 100 μl NLF using the PMN-Elastase ELISA according to the manufacturer’s instructions (eBioscience, No. BMS269). An automated washer (SLT Typ Columbus, Labtechnologies, Austria) was used to wash plates and a FluoStar Galaxy spectrometer (BMG Labtechnologies, Offenburg, Germany) was used for detection.

### Body Mass Index (BMI)

Our study population includes children and adults. For children and adolescents, the WHO classification of underweight, normal and overweight are not suitable. Moreover, for people with chronic diseases leading to malnutrition and delayed growth, the usage of BMI can be problematic. Therefore, we used the BMI SDS_LMS_, which matches size, weight, age and gender and allows the comparison of children, adolescents and adults within one study [32,33].

### Statistical analysis

Experimental data were evaluated with SPSS 19 (IBM, Ehningen, Germany), MS Excel (Redmont, USA) and GraphPad Prism 5 (LaJolla, USA). Descriptive statistics of cytokines were expressed as a median ± range for patients and healthy controls. Longitudinal values of cytokines were compared using Wilcoxon Test for matched pairs. Comparison with healthy controls was performed using the Mann–Whitney U-Test and Fisher’s Exact test. Correlations between the measured cytokine values and clinical or serological parameters were calculated using Spearman’s Rho. Bonferroni Alpha correction was performed for all parameters tested with Spearman, Fisher’s Exact test and Mann–Whitney U. We tested for seven inflammatory markers and total cell count. *P*-values of these analyses were multiplied by the number of applied tests. *P*-values of < 0.05 were considered statistically significant.

## Results

### Demographic data

The mean age of the 17 CF patients (10 females and seven males) was 22.7 years (range 7–39, SD 8.2). Nine patients were homozygous for the CF mutation F508del, and eight patients were heterozygous. Further class 1–3 mutations (394delTT, M1303K, G551D, 2183AA > G) were found in four patients and class 4–5 mutations (R347P, 2789 + 5G > A) were also present in four patients. The median hospitalisation time was 6 days (range 2–14), and i.v. therapy was continued in most patients as home treatment for a total of 14 days.

The 52 healthy controls were within the range of 9–60 years old, with a mean age of 31.9 years (SD 13.7; 36 females and 14 males), and were used for a previously published study [14].

The second sample was gathered within a median of 6 days after beginning i.v. therapy (range 2–14 days). In the majority of patients, AB therapy was initialised in hospital and continued at home for a total duration of 14 days. It was directed against *P. aeruginosa* (in 15/17 patients), *S. aureus* or *S. aureus + H. influenzae* (one patient each) (see Tables 2 and 3 for therapy details). Demographic and serological data and clinical characteristics of the included patients are shown in Tables 2 and 3.

**Table 2:** Clinical and serological characteristics of included patients (nominal variables)

**Nominal variables**	**n**	**Absolute frequency**		
Gender (f/m)	17	10 (53.3%)/7 (47.7%)		
Body Mass Index (BMI) scoring^*1^	17	3 (17.7%)/3 (17.7) /11 (64.7%)/0 (0%)		
Severe under-/under-/normal-/overweight				
Pancreatic insufficiency	17	13 (76.5%)		
Diabetes mellitus	17	6 (35%)		
Allergy	17	10 (59.0%)		
Chronic rhinosinusitis (CRS)	17	11 (65%)		
EPOS criteria for CRS at inclusion [28]:		No	Acute remittent	Chronic
Nasal blockage or obstruction or congestion		3 (17.6%)	2 (11.8%)	12 (70.6%)
Anterior or posterior nasal drip		2 (11.8%)	4 (23.5%)	11 (64.7%)
Facial pain or pressure		15 (88.2%)	1 (5.9%)	1 (5.9%)
Reduction or loss of smell		12 (70.6%)	0	4 (23.5%)
Allergic rhinitis	17	7 (41%)		
Allergic Bronchopulmonary Aspergillosis (ABPA)	17	2 (11.8%)		
History of nasal surgery	17	6 (35%)		
Therapy	17			
Current azithromycin		13 (77%)		
Current steroids nasal		4 (24%)		
Recombinant DNase		8 (47%)		
i.v. ABs (twice per day, mg/kg):				
Ceftazidim (200)/tobramycin (10)		11 (65%)		
Tobramycin (10)/meropenem (100)		5 (29%)		
Ceftazidim (200)/tobramycin (10)/meropenem (100)		1 (5.9%)		
Permanent colonisation LAW with^*2^:		15 (88%)		
*P. aeruginosa* (mucoid)	17	13 (76.5%)		
*P. aeruginosa* (non-mucoid)		4 (23.5%)		
*S. aureus*		7 (41%)		
Permanent/intermittent colonisation UAW with^*3^:		7 (41.2%)/5 (29.4%)		
*P. aeruginosa* (mucoid)		7 (41.2%)		
*P. aeruginosa* (non-mucoid)		13 (76.5%)		
*S. aureus*		3 (17.7%)/ 4 (23.5%)		
*P. aeruginosa* serum antibody positive:	15			
Alcaline protease		3 (20%)		
Elastase		6 (40%)		
Exotoxine A		4 (27%)		

^*1^: *SDSLMS*: Standard-Deviation-Score; *L* = Box cox-power transformation, *M* = median, *S* = variation coefficient.

^*1^: BMI scoring was carried out according to [33].

^*2^: Permanent and intermittent colonisation was stated using the criteria published by Lee et al. [31], where the authors define a chronic colonisation if more than 50% of cultures within the last year were found to be positive and intermittent if less than 50% of cultures within the last year were found to be positive.

**Table 3:** Clinical and serological characteristics of included patients (metric and ordinal variables)

**Metric and ordinal variables**	**n**	**Mean**	**SD**	**Median**	**Range**
Age (yrs.)	17	22.7	8.2	22.0	7-39
Weight (kg)	17	51.7	14.6	51.4	22.0-73.3
Height (cm)	17	160.9	15.57	162	117.8-180
BMI (kg/m^2^)	17	19.5	3.2	20.4	12.6-24.2
SDS_LMS_^*1^	17	-1.2	1.3	-1.1	-5.5-0.1
ESR after 1/2 h (mm/h)	13	21.2/42.3	18.1/28.7	18/41	1-61/9-104
CRP (mg/l)	17	12.5	13.8	5.6	2.0-47.0
FEV1 (l)/(% predicted)	17	1.9/62.7	0.8/24	1.7/65.5	0.8-3.9/24.3-108.6
MEF75/25 (l)/(% predicted)	15	1.7/46	2.03/39	1.1/30	0.3-7.9/7.4-153.3
Shwachman Score (without chest X-ray)	17	68.2	10.0	70.0	35.0-75.0
Total IgE (KU/l)	17	249.7	481.1	76.8	1.9-1944.0
Total IgG (KU/l)	17	13.8	4.1	13.3	5.9-19.5
Retrieved NL volume prior therapy (mL)	15	11.5	2.0	12.0	8.0-16.0
Retrieved NL volume during/after therapy (mL)	15	11.3	1.19	11.0	10.0-13.0

### Serological data

Erythrocyte sedimentation rate (ESR) was elevated in 85% of patients (11/13, range 1 h 1–61 mm/h, 2 h 9–104 mm/h) and CRP in 41% (7/17, median 5.60 mg/L, range 2–47 mg/L). IgG was elevated in 35% of patients (6/17, median 13.30 g/L, range 5.85–19.50 g/L), IgA (median 1.84 g/L, range 0.07–6.60 g/L) and IgE (median 76.80 KU/L, range 1.90–1944.00 KU/L) in 5/17 patients (29.4%). IgM was within the normal range in all 17 patients (median 1.23 g/L, range 0.67–2.01 g/L). Fibrinogen, as a marker for chronic inflammation, was elevated in 3/17 patients (21%, median 2.90 g/L, range 2.20–4.30 g/L).

### Methodological issues

#### NL recovery

**Figure 1:**
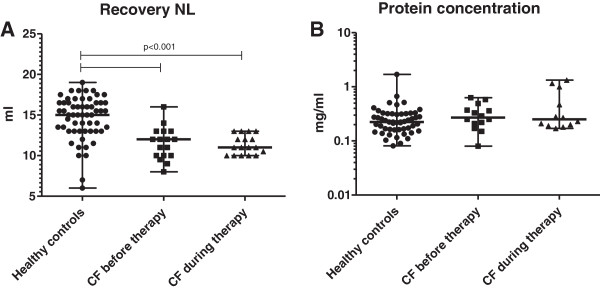
**Differences in NLF recovery volumes (A) and protein concentrations (B) between healthy controls and CF patients, before and during i.v. AB therapy.** For NL, each nostril was rinsed with 10 mL of isotonic saline (total volume: 20 mL).

NL backflow volume did not differ in CF patients prior to and during therapeutic intervention (median 12.0 mL, range 8–16 mL and 11.0 mL, range 10–13 mL, respectively, *P* = 0.62). However, in healthy controls, the recovery was slightly higher (median, 15.0 mL, range 6–18 mL, *P* < 0.0001, Figure 1A).

#### Protein concentration

The median NLF protein concentration was 0.25 mg/mL (range 0.08–0.63 mg/mL) in CF patients prior to therapy and 0.27 mg/mL (range 0.17–1.33 mg/mL) for CF patients during therapy. The median NLF protein concentration of the controls was 0.22 mg/mL (range 0.08–1.70 mg/mL). Standardisation of analyte concentrations to protein concentration did not change the significance levels of the results (see Figure 1B).

### Cytological analysis

**Figure 2:**
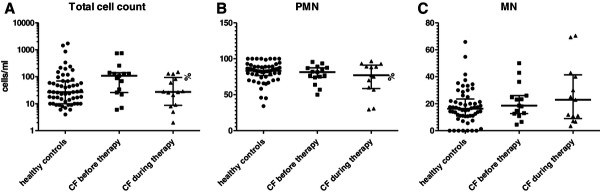
Differences in NLF total cell counts (A), polymorphonuclear neutrophils (PMN) (B) and mononuclear cells (MN) (C) between healthy controls and CF patients, before and during i.v. AB therapy.

The total cell count was lower in healthy controls (median 27 cells/mL, range 0–1723 cells/mL) than in untreated CF patients (median 108 cells/mL, range 6–744 cells/mL), although the differences did not reach statistical significance (*P* = 0.088). After 6 days of i.v. AB therapy, the median total cell count decreased to a level comparable to that in healthy controls (median 28 cells/mL, range 5–150 cells/mL; *P* = 0.104, Figure 2A). As shown in Figure 2B and 2C, some non-significant trends were seen in the distribution of cell types. Total PMN and mononuclear cell (MN) counts were lower in healthy controls compared with CF patients before and during i.v. AB treatment. The proportion of PMNs of total leukocytes was higher in healthy controls compared with CF before and during therapy. Conversely, the proportion of MN was lower in healthy controls compared with CF.

### Comparison of inflammatory markers in healthy controls and CF patients prior to and during i.v. AB therapy

**Figure 3:**
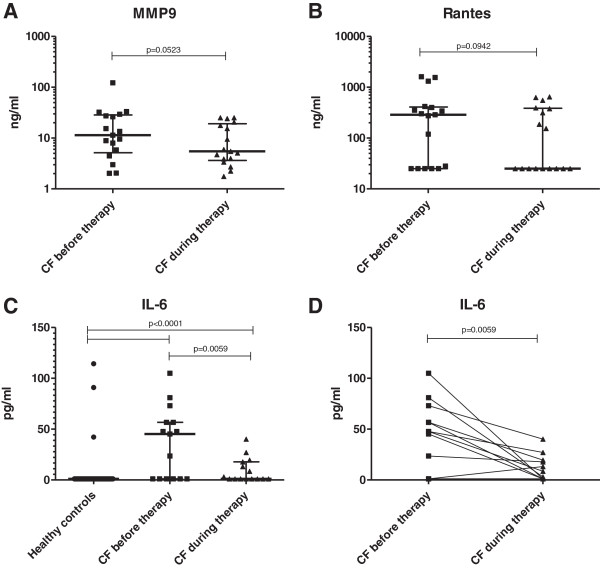
**Differences in inflammatory markers in healthy controls and CF patients, before and during i.v. antibiotic therapy.** Matrixmetalloproteinase 9 (MMP9) was clearly attenuated during therapy **(A)**. RANTES levels declined under AB treatment **(B)**. IL-6 was found to be significantly elevated in CF **(C)**, declining during antibiotic treatment in almost all CF patients **(D)**.

Detection frequency and concentrations of all measured inflammatory markers were significantly higher in CF patients compared with healthy controls (see Table 1). During AB therapy the median detection frequencies of IL-8, IL-6, IL-1β and RANTES decreased, while MMP9, MPO and NE detection frequencies did not change. For TNF, a slight increase in detection frequency during treatment was found. When the concentration of inflammatory markers was analysed, a decline in all the analysed parameters except TNF was observed. Notably, a strong decrease in IL-6 was found in 16/17 CF patients during AB therapy (*P* = 0.0059, Table 1 and Figure 3C and 3D) and also in MMP9 and RANTES, where the median values declined two-fold and 10-fold, respectively (Table 1, Figure 3A and 3B and Additional file 1: Figure S1).

### Correlations between inflammatory markers during i.v. AB therapy

MMP9 in NL correlated significantly with MPO both before and during treatment (r = 0.559, *P* = 0.020, and r = 0.578, *P* = 0.013, respectively, data not shown). Before AB therapy, there was also was a significant positive correlation between MPO and TNF (r = 0.684, *P* = 0.005).

IL-8 levels in NL before treatment correlated with IL-8 levels during AB therapy in a highly significant manner (r = 0.719, *P* = 0.001). Furthermore, IL-8 correlated with IL-1β, both before and after therapy (r = 0.658, *P* = 0.008, and r = 0.646, *P* = 0.009, respectively). IL-6 correlated with MMP9 before and during treatment (r = 0.564, *P* = 0.029, and r = 0.630, *P* = 0.007) and RANTES (r = 0.744, *P* = 0.001 and *P* = 0.001, r = 0.797).

TNF correlated with RANTES both before and during therapy (r = 0.676, *P* = 0.006 and, r = 0.870, *P* = 0.001). During i.v. AB treatment, higher values of TNF were associated with higher values of IL-1β (r = 0.625, *P* = 0.013) and with lower concentrations of NE (r = -0.578, *P* = 0.031). NE concentrations in NL before and after therapy showed a significant correlation within the same patient (r = 0.524, *P* = 0.037).

### Correlation between inflammatory markers and clinical parameters

Systemic inflammation evaluated by CRP and ESR, as well as lung function parameters, was only measured prior to i.v. treatment. We did not find a significant correlation between systemic inflammation markers (CRP and ESR) and inflammatory mediators measured in NL. Moreover, some trends were seen for IL-1β that showed a negative correlation to CRP.

At the starting point of the longitudinal study we found no significant correlations between FEV1 (% predicted) and cytokine concentrations, except for MMP9 and IL-1β, which revealed a trend for a positive correlation.

## Discussion

The present study describes, for the first time, changes in cytokine expression and cytological dynamics in the NLF of CF patients during i.v. AB intervention.

We have demonstrated that the total cell count in NL, which was significantly increased in CF patients compared with healthy controls, declined to normal levels during a median time of 6 days of systemic AB treatment. This corresponds well with findings from the lower airways (BAL) as previously reported [34]. Accordingly, the absolute numbers of PMNs and MNs are lower in healthy controls compared with CF patients. Indeed, the percentages of PMN vs. MN were slightly different, but not significantly so, between CF patients and healthy controls and no change in the percentage was observed during AB treatment. This may support the results of Johansen et al. [35], who found a reduced PMN response, but elevated non-inflammatory secretory IgA levels, on *P. aeruginosa* biofilms colonising CF patients’ upper airways. Furthermore, the level of IgA can discriminate between non-, intermittent and chronically colonised patients with the high concentration of IgA in the last group being used as a diagnostic tool [36]. On this basis, the authors hypothesise that impaired sinonasal PMN recruitment gives rise to a failure to eradicate *P. aeruginosa* from the upper airway segment by the immune system. Moreover, histological studies have revealed an enhanced presence of mast and plasma cells in sinonasal tissue from CF patients [37]. Our results also correspond to those from sputum [38], where AB treatment had no influence on total or differential cell count in CF lower airway secretions, which is in contrast to the expected reduction of inflammation with therapeutic reduction of pathogens.

The inflammatory markers MPO, IL-8, IL-6 and IL-1β were found significantly more often in NLF of CF patients, when compared with healthy controls. This is in line with previous findings from retrospective studies [14]. In addition to the aforementioned cytokines, TNF concentration in the upper airway lavage was found to be higher in CF patients compared with controls. For LAW sampling by BAL, Elizur et al. [39] reported similar results. Furthermore, the median expression levels of MPO, IL-8, IL-6, and IL-1β in NLF were significantly higher in our CF cohort compared with healthy controls.

During i.v. therapy detection the frequency of IL-8, IL-6, IL-1β and RANTES decreased. Simultaneously, IL-6 levels in NLF declined significantly. RANTES and MMP9 decreased to a lower, but not significant, extent. Notably, the changes of IL-6 in NL are in accordance with the recent publication of Paats et al. [40]. In this study, UAW inflammation in CF patients was assessed during and approximately 3 months after airway exacerbation. In contrast, our study focused on the change of UAW inflammation after only 6 days of elective IV antibiotic treatments. In this regard, the results from Paats et al. and our study underline that IL-6 is a highly sensitive biomarker for infection and antibiotic effects in non-invasively sampled airway secretions from the upper airways.

Additionally, no changes in the expression levels of NE, which was detected for the first time in NLF, were observed during the applied therapeutic intervention. Also TNF, MPO, IL-8 and IL-1β remained unchanged during 6 days of therapy. We cannot differentiate whether this is due to the relatively short period of systemic AB treatment or the above-mentioned reduced PMN response in the upper airways, as suggested by Johansen et al. who hypothesised the presence of different defence mechanisms in the upper and lower airways [35]. This is in line with data from our recent study, where higher neutrophil counts and IL-8 levels were detected in sputum compared with nasal lavage fluid [41]. Moreover, the hypothesis of differences in host defence mechanisms between the upper and lower airways was also supported by data from a current work from Michl et al. Nasally exhaled nitric oxygen (NO), which is a first-line defence mechanism in paranasal sinuses, was significantly reduced in CF patients with elevated CRP and ESR, an effect not seen in the LAW [42].

Interestingly, there is a strong correlation between different inflammatory markers. MMP9 levels were associated with MPO and IL-6 levels both prior to and during therapy. Furthermore, RANTES levels are associated with TNF and a positive correlation was found to exist between IL-1β and IL-8 prior to and during therapy. We analysed protein-protein interactions in-silico, using the online databank string-db.org (http://string-db.org/). Additionally, other publications have shown a correlation between MPO and MMP9 [43], but we did not find a direct interaction between them. It is postulated that MPO activates MMP9, which is released in an inactive form [44]. As MPO plays an important role during oxidative bursts, a correlation with other pro-inflammatory markers may be due to increased Reactive Oxygen Species (ROS). Expression of MMP9 is induced by IL-1β, IL-6 or TNF [45,46], and IL-1β increases IL-6 expression [47]. IL-1β is degraded by MMPs, a process that can be blocked by TIMP-1 [48]. For IL-6 and RANTES no direct interactions were listed, but both markers are elevated in tissues infected with *P. aeruginosa*[49] and during acute pulmonary exacerbation in CF patients [50]. An interaction between IL-1β and IL-8 has been described previously: IL-1β stimulates IL-8 expression [51], and binds and activates IL-8. ROS stimulates the release of IL-1β and TNF, which leads to enhanced detachment of IL-8, IL-6, MMP9 and TNF (e.g. via NFκB or Mitogen-Activated-Protein-Kinase (MAPK)) [52]. Bacterial infections lead to NFκB activation via Toll-like receptors in airway epithelial cells and alveolar macrophages or dendritic cells, which in turn induce transcription of pro-inflammatory cytokines such as IL-6 and IL8. Furthermore cytokines, such as IL-1β and TNF, can activate NFκB, which seems to be a key factor in NLF inflammation signalling. NFκB is inhibited by macrolide AB-like azithromycin (AZM). Seventy per cent of our patients received AZM as anti-inflammatory therapy. In these patients we found lower IL-1β levels when compared with untreated patients (Additional file 1: Figure S2), but because of the small proportion of untreated patients, the observed differences may not be representative. Therefore, in future studies, it would be of great interest to evaluate the NFκB levels and activity in airway epithelial cells.

The present study did not reveal significant correlations between systemic inflammation and inflammatory marker concentrations in NL, which accords well with the hypothesis of a compartmentalised infection and inflammation in CF. Moreover, we found no correlation between lung function and CRS status. This may be due to the small size of the study cohort. As a result of the short period of time and the wide range of duration that patients stayed in the hospital after the initialisation of i.v. therapy, we did not collect systemic inflammatory markers and lung function data during therapy. The heterogeneity of the investigated patients regarding age, pulmonary function and nutritional status is compensated for by the longitudinal nature of the study, as assessing changes in nasal inflammatory markers during intravenous AB treatment was its principal aim. Therefore, future studies should assess larger patient cohorts for longer periods and include NL and LAW assessment at the end of therapy, and if possible, assessment of systemic inflammatory markers and microbiology.

NL may be of interest for other systemic and topical therapeutic approaches in CF, and also other respiratory diseases including allergic rhinitis/allergic asthma and immune deficiencies. Moreover, microbiological and inflammatory marker assessment of NLF can provide information about the prevalence and impact of compartmentalised airway infection in various respiratory diseases, for example ventilator-associated pneumonia and sepsis.

## Conclusions

In contrast to BAL, nasal lavage is a non-invasive method and allows for the frequent sampling of airway surface liquid. In the present study, we found substantial differences in longitudinally collected NLF from CF patients, both before and after a median of 6 days of i.v. AB treatment, and compared with healthy individuals. Substantial differences between the three groups were evident after only this short period of therapy. Total NL cell counts, initially elevated in CF, decreased to the level of healthy controls. IL-6 was significantly reduced, with a trend towards reduced RANTES and MMP9. The latter, together with NE, were assessed in the NL of CF patients for the first time. Our findings highlight the use of NL as a potential tool for clinical and scientific studies.

## Abbreviations

CF: Cystic Fibrosis; CFTR: Cystic Fibrosis Transmembrane Conductance Regulator; NLF: Nasal lavage fluid; NL: Nasal lavage; BAL: Brochoalveolar lavage fluid; UAW: Upper airways; LAW: Lower airways; TCC: Total cell count; PMN: Polymorphonuclear; MN: Mononuclear; NE: Neutrohil elastase; IL: Interleukin; MMP: Matrix metalloproteinase; TIMPs: Tissue inhibitors of metalloproteinases; MPO: Myeloperoxidase; TNF: Tumor necrosis factor; IFN: Interferon; RANTES: Regulated on activation, normal T cell expressed and secreted; i.v.: Intraveneuos; AB: Antibiotic; SDSLMS: Standard-Deviation-Score; L: Box cox-power transformation; M: Median; S: Variation coefficient; LTx: Lung transplantation.

## Competing interest

We confirm that there are no known conflicts of interest associated with this publication and there has been no external funding received for this study that could have influenced its outcome.

## Author’s contributions

JR supervised the experimental section and wrote the manuscript mainly. MJ, NB, NF and FD collected patients’ material and carried out the experiments. RM, UM, MP and JM critical revised the manuscript. KB performed the cytological and PK the microbiological analyses. TL worked as statistical counsellor. All authors read and approved the final manuscript.

## Pre-publication history

The pre-publication history for this paper can be accessed here:

http://www.biomedcentral.com/1471-2466/14/82/prepub

## Supplementary Material

Additional file 1: Figure S1 Differences in inflammatory markers in healthy controls and CF patients, before and during i.v. antibiotic therapy. Significant differences in myeloperoxidase between CF and healthy controls were observed and a slight decline under AB intervention was found (S1A). IL-1β (S1B) and IL-8 (S1C) levels were significantly lower in controls than in CF patients. TNF was significantly elevated in CF patients (S1D), but there was no change during AB treatment. No changes in NE were observed under AB treatment (S1E). **Figure S2.** Lower IL-1β levels were observed in AZM-treated patients (median 140.6 ng/mL, range 4.1–467.2) compared with untreated patients (747.1 ng/mL, range 219.5–779.3, *P* = 0.0348). Click here for file
