# Supplementary material for: Dynamics of soluble and cellular inflammatory markers in nasal lavage obtained from Cystic Fibrosis patients during intravenous antibiotic treatment
Source: BMC Pulm Med. 2014 May 13;14:82. doi: 10.1186/1471-2466-14-82 (PMC4024110; doi:10.1186/1471-2466-14-82)
Supplement: Additional file 1: Figure S1 — Differences in inflammatory markers in healthy controls and CF patients, before and during i.v. antibiotic therapy. Significant differences in myeloperoxidase between CF and healthy controls were observed and a slight decline under AB intervention was found (S1A). IL-1β (S1B) and IL-8 (S1C) levels were significantly lower in controls than in CF patients. TNF was significantly elevated in CF patients (S1D), but there was no change during AB treatment. No changes in NE were observed under AB treatment (S1E). Figure S2. Lower IL-1β levels were observed in AZM-treated patients (median 140.6 ng/mL, range 4.1–467.2) compared with untreated patients (747.1 ng/mL, range 219.5–779.3, P = 0.0348). [file 1471-2466-14-82-S1.ppt]

## Slide 1
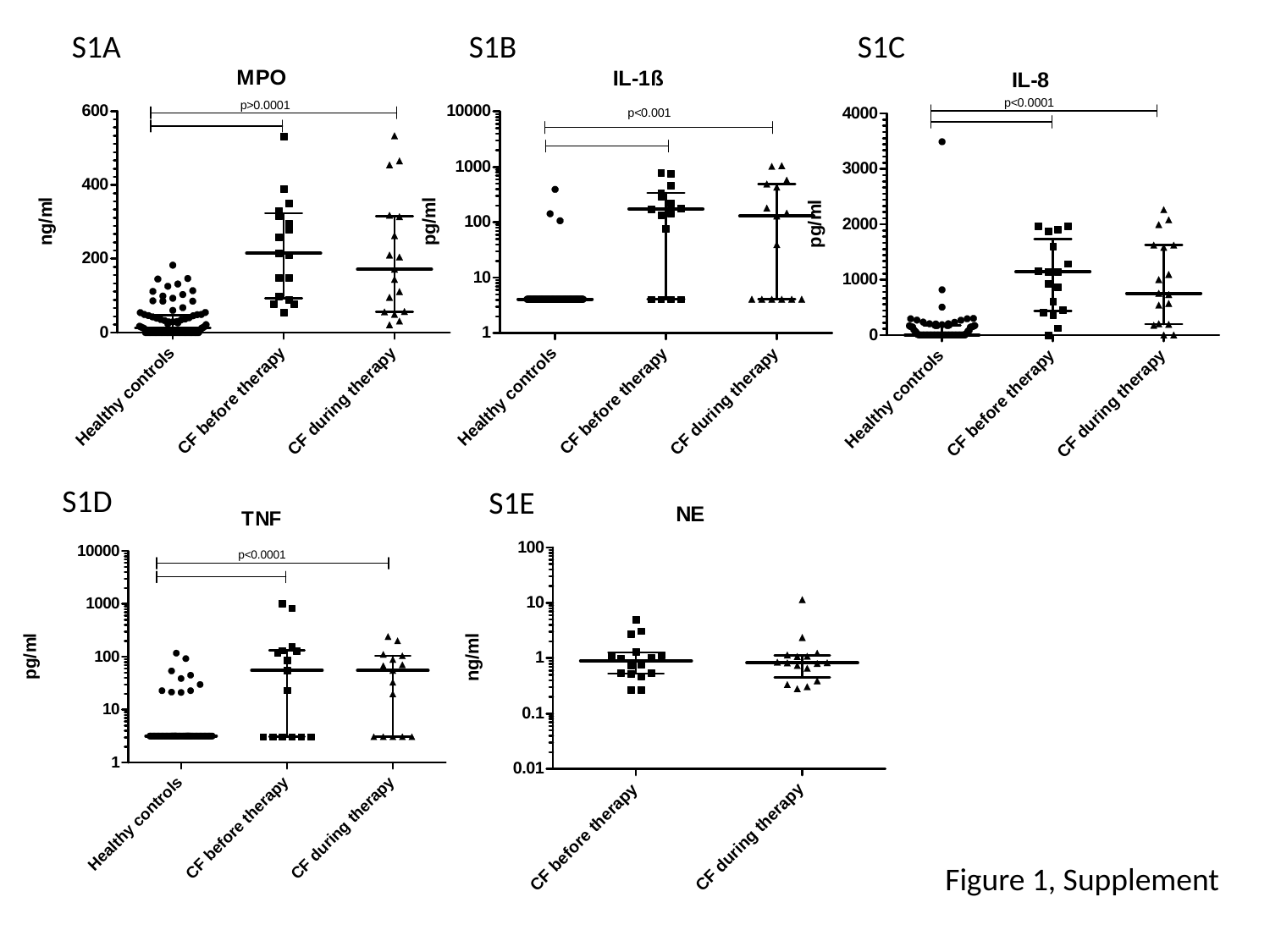

S1A
S1B
S1C
S1D
S1E
Figure 1, Supplement

## Slide 2
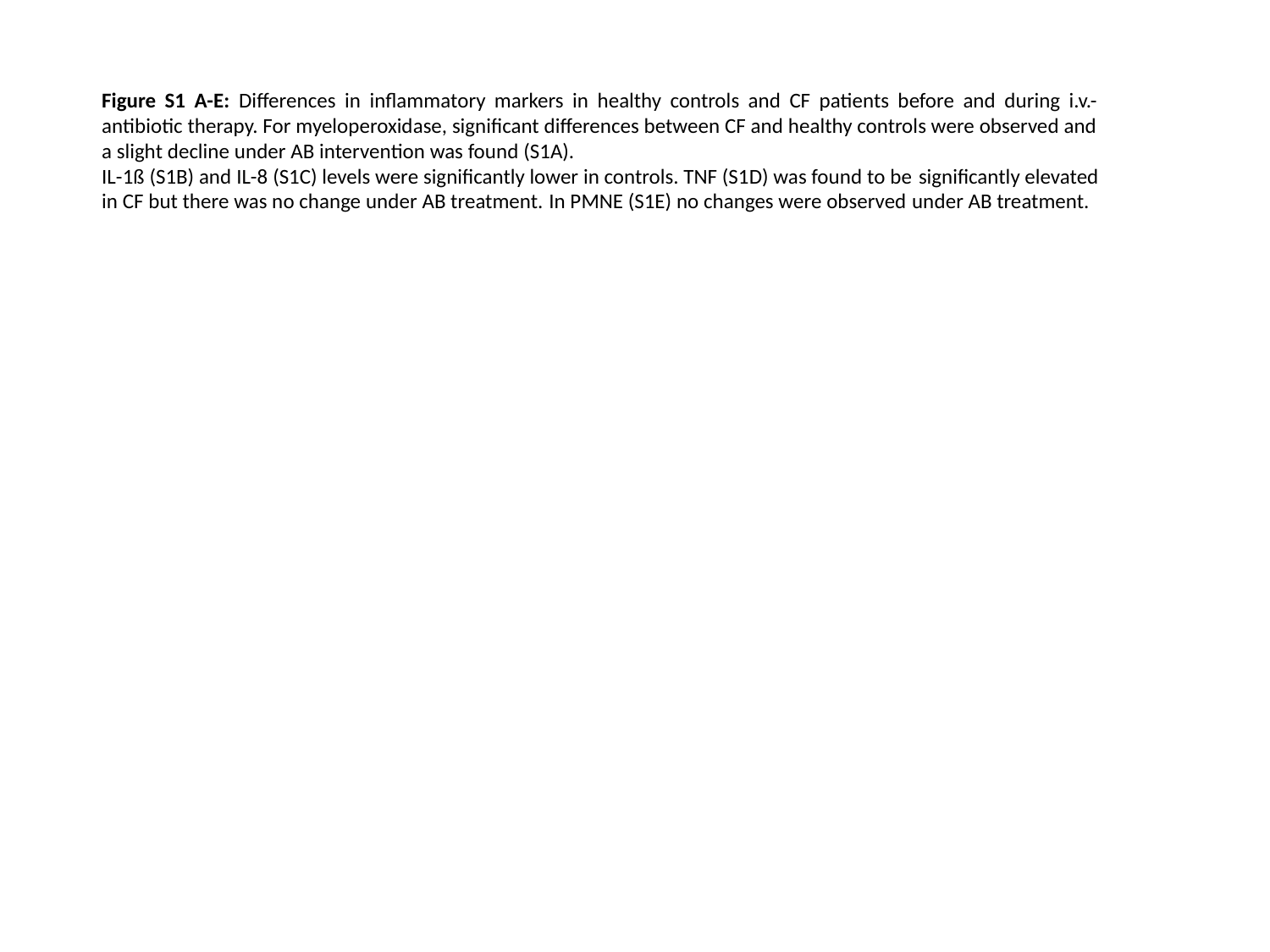

Figure S1 A-E: Differences in inflammatory markers in healthy controls and CF patients before and during i.v.-antibiotic therapy. For myeloperoxidase, significant differences between CF and healthy controls were observed and a slight decline under AB intervention was found (S1A).
IL-1ß (S1B) and IL-8 (S1C) levels were significantly lower in controls. TNF (S1D) was found to be significantly elevated in CF but there was no change under AB treatment. In PMNE (S1E) no changes were observed under AB treatment.

## Slide 3
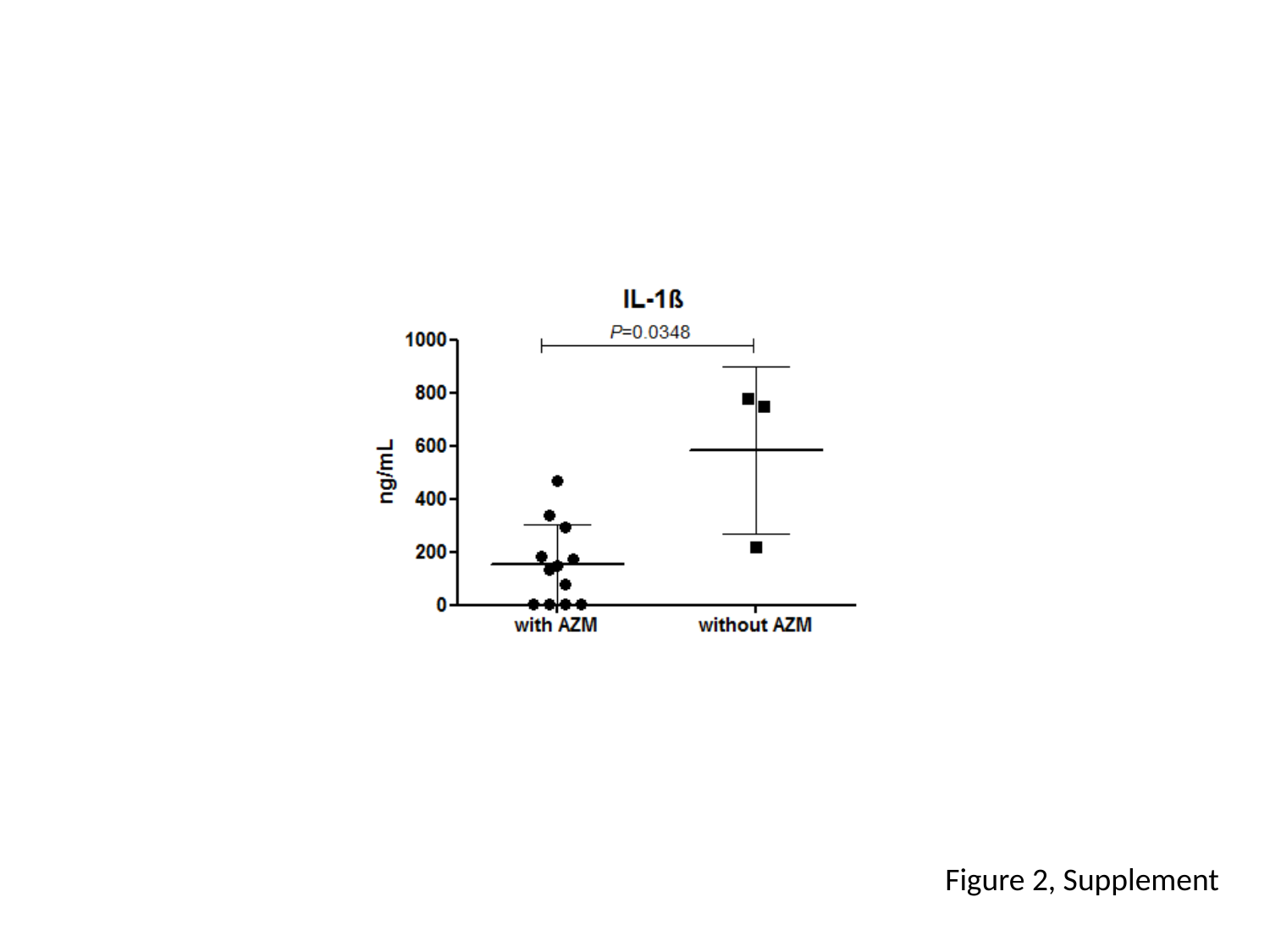

Figure 2, Supplement

## Slide 4
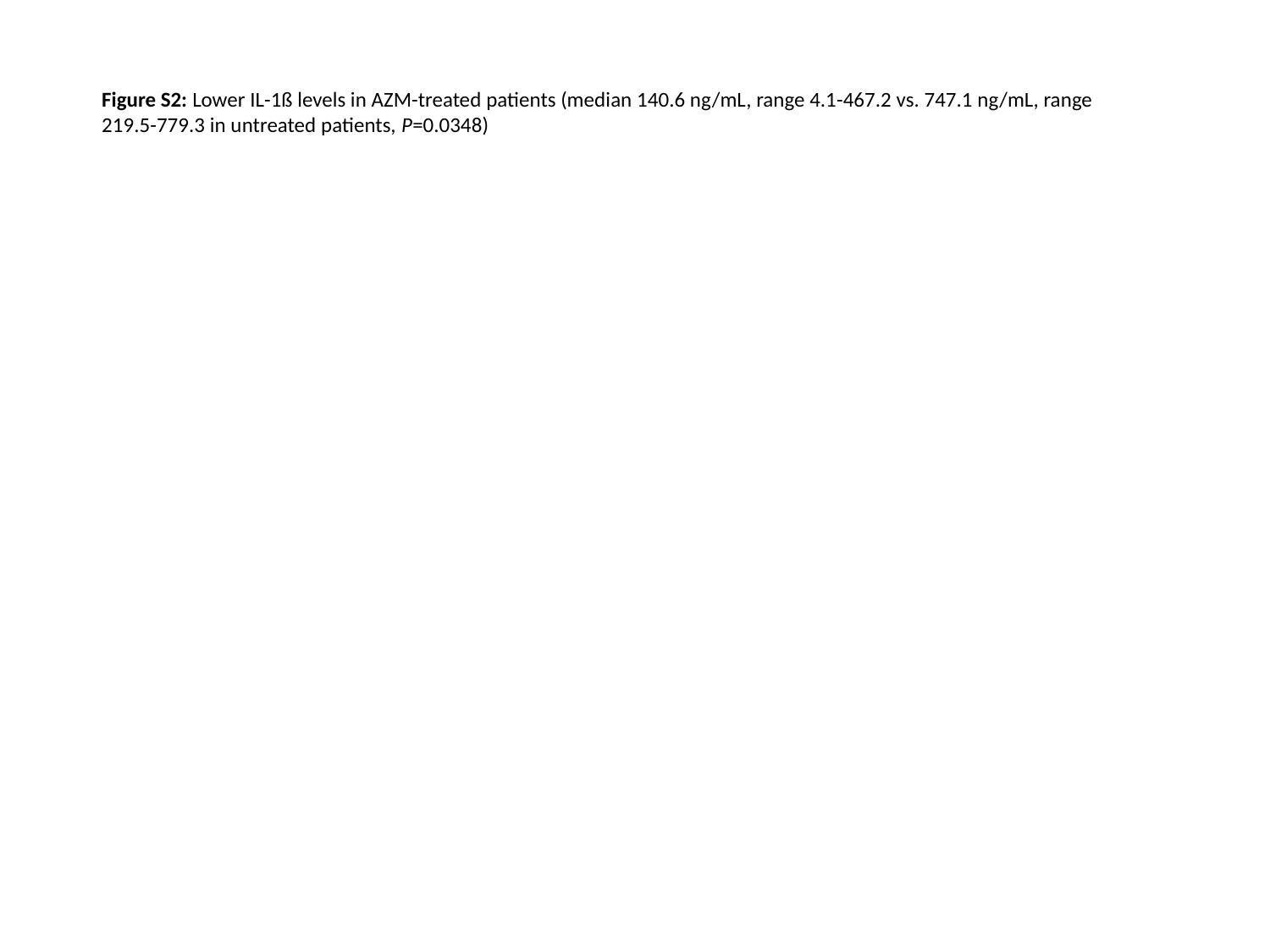

Figure S2: Lower IL-1ß levels in AZM-treated patients (median 140.6 ng/mL, range 4.1-467.2 vs. 747.1 ng/mL, range 219.5-779.3 in untreated patients, P=0.0348)
